# Ceragenin CSA-44 as a Means to Control the Formation of the Biofilm on the Surface of Tooth and Composite Fillings

**DOI:** 10.3390/pathogens11050491

**Published:** 2022-04-20

**Authors:** Joanna Tokajuk, Piotr Deptuła, Sylwia J Chmielewska, Karol Skłodowski, Żaneta A Mierzejewska, Małgorzata Grądzka-Dahlke, Adam Tołstoj, Tamara Daniluk, Paulina Paprocka, Paul B Savage, Robert Bucki

**Affiliations:** 1 Department of Medical Microbiology and Nanobiomedical Engineering, Medical University of Białystok, Białystok, 15-222 Bialystok, Poland; asiatokajuk@gmail.com (J.T.); piotr.deptula@umb.edu.pl (P.D.); sylwia.chmielewska@umb.edu.pl (S.J.C.); karol.sklodowsky@gmail.com (K.S.); tamara.daniluk@umb.edu.pl (T.D.); 2 Dentistry and Medicine Tokajuk, Żelazna 9/7, 15-297 Bialystok, Poland; 3 Institute of Biomedical Engineering, Faculty of Mechanical Engineering, Białystok University of Technology, Wiejska 45C Street, 15-351 Białystok, Poland; a.mierzejewska@pb.edu.pl; 4 Institute of Mechanical Engineering, Faculty of Mechanical Engineering, Białystok University of Technology, Wiejska 45C Street, 15-351 Białystok, Poland; mgdahlke@gmail.com (M.G.-D.); a.tolstoj@pb.edu.pl (A.T.); 5 Institute of Medical Science, Collegium Medicum, Jan Kochanowski University of Kielce, IX Wieków Kielc 19A, 25-317 Kielce, Poland; paulina.paprocka@ujk.edu.pl; 6 Department of Chemistry and Biochemistry, Brigham Young University, Provo, UT 84602, USA; pbsavage@chem.byu.edu

**Keywords:** ceragenin, CSA-44, *Candida albicans*, *Enterococcus faecalis*, biofilm eradication, dental composite

## Abstract

Recurrent oral infections, as manifested by endodontic and periodontal disease, are often caused by *Enterococcus faecalis* (*E. faecalis*) and *Candida albicans* (*C. albicans*). Here, we assessed the anti-biofilm activity of ceragenin CSA-44 against these microbes growing as a biofilm in the presence of saliva on the surface of human teeth and dental composite (composite filling) subjected to mechanical stresses. **Methods:** Biofilm mass analysis was performed using crystal violet (CV) staining. The morphology, viscoelastic properties of the biofilm after CSA-44 treatment, and changes in the surface of the composite in response to biofilm presence were determined by AFM microscopy. **Results:** CSA-44 prevented biofilm formation and reduced the mass of biofilm formed by tested microorganisms on teeth and dental composite. **Conclusion:** The ability of CSA-44 to prevent the formation and to reduce the presence of established biofilm on tooth and composite filling suggests that it can serve as an agent in the development of new methods of combating oral pathogens and reduce the severity of oral infections.

## 1. Introduction

Biofilm is an organized structure consisting mainly of bacteria, possibly fungi, and protozoa, which bonds firmly to the colonized surface [1,2]. Dental biofilm formed by physiological microflora may be beneficial because it hinders colonization by exogenous, unfavorable microorganisms or pathogens. On the other hand, changes in the microflora composition of the biofilm (dysbiosis) may result in disease development [3]. Oral infections, manifested by endodontic and periodontal disease, can be caused by *C. albicans* and *E. faecalis*. Propagation of dental biofilm may cause diseases in the oral cavity, including dental caries, pulpitis, and apical periodontitis [3,4,5,6,7,8]. Bacteria colonize every surface in the oral cavity: teeth, dental fillings, fixed and removable prosthetic restorations, fixed orthodontic appliances, and mucosa. Dental plaque on the teeth is formed by ecological succession: early colonizers adhere weakly to pellicle-covered enamel [3], followed by stronger adhesion through glycoprotein receptors and bacterial surface adhesins [3,9]. Initial colonizers are often Gram-positive streptococci, followed by Gram-positive rods, and then other Gram-positive bacteria. Biofilms that are formed on dental fillings can destroy the structure of the filling components, which results in a rougher surface of the material [10], and rough surfaces are more susceptible to colonization [10,11]. Bacteria can penetrate between the filling components and the tooth structure [12], which may cause secondary caries [13] and pulp inflammation. During maturation of the biofilm, microbes are metabolically active, and they use endogenous components from saliva to form an extracellular matrix that helps with co-adhesion [14]. In a fully-fledged biofilm, bacteria reduce metabolic activity and become less susceptible to eradication by the host’s immune system, to mechanical removal [14,15], and to the action of microbicidal agents [16]. In addition, biofilm subpopulations on dental materials can trigger gene expression, resulting in increased resistance to antibiofilm molecules. There are reports of agents added to dental materials that inhibit biofilm formation or kill the bacteria within its structure, such as silver, zinc oxide and bioglass particles, MDPB monomers, and nanoparticles of quaternary ammonium polyethylenimine (QPEI) [9]. Over time, most of these undergo chemical and mechanical degradation, which reduces their effectiveness [17]. Accordingly, other antimicrobials and nanosystems are being sought to inhibit biofilm growth or biofilm degradation.

In our study, we assessed an antimicrobial from the ceragenin family of compounds, termed CSA-44 (cationic steroid antibiotics), for the prevention of biofilm formation and eradication of an existing biofilm. As with other ceragenins, CSA-44 mimics the activity of natural antimicrobial peptides (AMPs) that function as effector molecules of the innate immune system and have broad-spectrum antimicrobial activity. Bacteria, in general, are susceptible to this class of antimicrobials, and wide-spread resistance to those molecules has not been reported. AMPs and ceragenins associate with phospholipids in bacterial membranes, resulting in a change in the structure of the membranes and causing depolarization. Because ceragenins are non-peptide bases, they are insensitive to proteolysis, which is an advantage over AMPs [18,19]. In addition to antibacterial activity, ceragenins display, antiviral, sporicidal, antiparasitic, and anticancer activities [19,20]. In a recent study, we showed that ceragenins decrease the adherence of biofilms on surfaces and display antimicrobial activity against bacteria and fungi associated with oral infections. These observations motivated us to evaluate the activity of CSA-44 (a ceragenin with high biocompatibility) against established biofilms developed on teeth and composite fillings and its ability to disrupt biofilm adhesion to those materials.

## 2. Results

### 2.1. Biofilm Mass Studies

**Figure 1:**
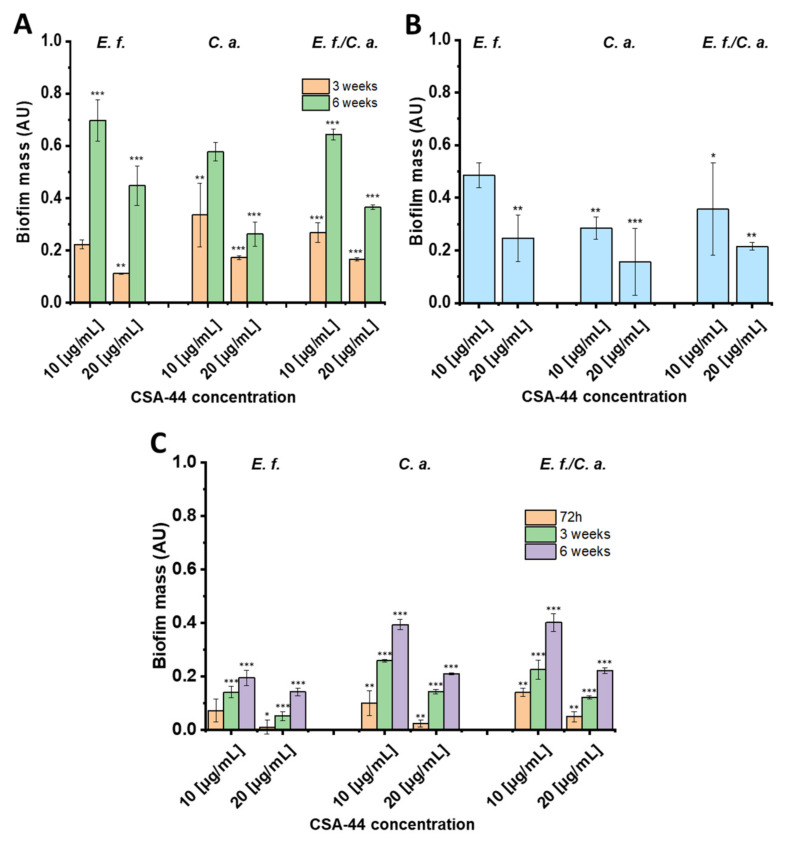
(**A**) Reduction of preformed biofilm mass of *Enterococcus faecalis* (*E. f.*), *Candida albicans* (*C. a.*), and *Enterococcus faecalis/Candida albicans (E. f./C. a.*), relative to untreated controls, on the surface of human teeth using CSA-44 in human saliva. (**B**) Prevention of biofilm formation on the surface of dental composites in the presence of CSA-44 during 72 h period of biofilm growth in saliva. CSA-44 was added to the culture medium for 2 h, then washed out, and CV staining was performed. (**C**) Reduction of preformed biofilm mass, relative to untreated controls, on the surface of composite fillings, using CSA-44 in saliva. For all plots, the relative weight of the biofilm was normalized to the original weight of the teeth and composite disks. The dye was absorbed by the samples themselves; the absorbance value of the negative control was subtracted from all the absorbance results obtained for the test samples and the positive control for each microorganism. The absorbance outcome for the positive control was considered as 100%. The results obtained for the test samples, i.e., under the treatment with CSA-44, were related to this value. Results show: mean ± SD from 3 measurements. * indicates statistical significance at ≤ 0.05, ** ≤ 0.01, and *** ≤ 0.001.

Biofilm of *Candida albicans* ATCC 26790 (*C. albicans*), *Enterococcus faecalis* ATCC 29212 (*E. faecalis*), and *Enterococcus faecalis* ATCC 29212 + *Candida albicans* ATCC 26790 (*E. faecalis/C. albicans*) on human teeth or dental composites was generated for 3- and 6-week time periods (for composite, a time period of 72 h was evaluated as well). For one set of experiments, biofilm was grown in the presence of CSA-44 on the composite material for 72 h to assess the ability of CSA-44 to prevent biofilm formation. The data demonstrate the comparison of the results between the untreated control and samples under the exposure to CSA-44. Figure 1A shows the reduction of 3- or 6-week biofilms on human teeth, measured with crystal violet staining, treated with the indicated concentrations of CSA-44 for 2 h in human saliva. The mass of 3-week biofilm, after exposure to CSA-44 (10 µg/mL), decreased by 66–78% compared to untreated control. This effect was even more pronounced after treatment with 20 µg/mL of CSA-44, where an 83–89% decrease in biofilm mass was observed. A 30–42% and 55–74% reduction in the 6-week biofilm mass was observed after exposure to CSA-44 (10 µg/mL and 20 µg/mL, respectively).

Prevention of biofilm formation was measured over 72 h by adding CSA-44 to dental composites and then inoculating (Figure 1B). CSA-44 inhibited biofilm formation on the dental composites, and the greatest antibiofilm activities of CSA-44 were observed with *C. albicans.* Compared with the untreated control, exposure to 10 µg/mL CSA-44 resulted in a decrease in *C. albicans* biofilm mass by 72%, and with 20 µg/mL CSA-44, the reduction of biofilm mass reached 84%. Prevention of biofilm formation recorded for *E. faecalis* and *E. faecalis*/*C. albicans* in response to 20 µg/mL of CSA-44 were 75 and 79%, respectively.

Panel 1C presents the disruption of preformed biofilm on polymer composite after 72 h and 3 and 6 weeks upon treatment with CSA-44 (2 h, RT; 10 or 20 µg/mL). With each microorganism, a significant decrease in the biofilm mass was observed with concentrations of 10 and 20 µg/mL. This effect was particularly evident with the 20 µg/mL concentration; with *E. faecalis* and 72 h biofilm, a reduction of approximately 99% of biofilm mass in relation to the untreated control was observed. The reduction of preformed biofilm mass after incubation with CSA-44 indicates that this ceragenin possesses the capacity to disrupt the mature biofilms in a concentration-dependent manner. Notably, the duration of biofilm pre-growth significantly impacted the reductions by both concentrations of CSA-44.

### 2.2. Microscopic Studies of the Surfaces of Teeth and Dental Composites

**Figure 2:**
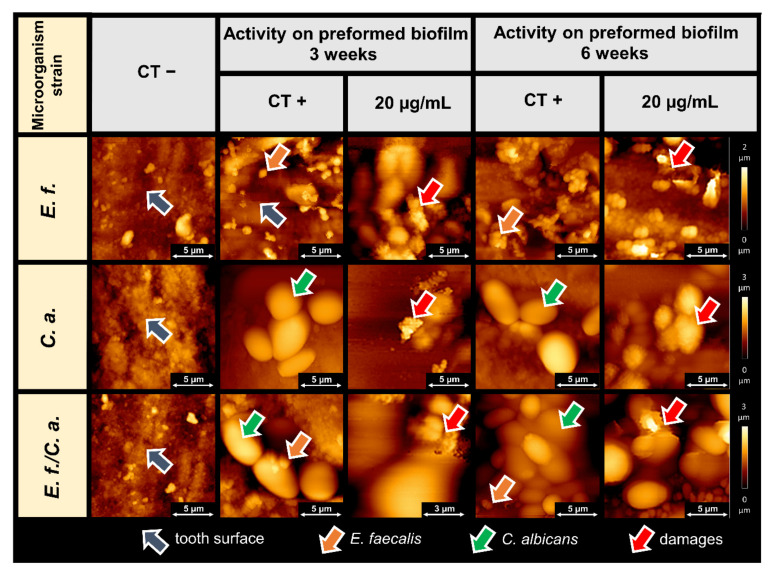
Atomic force microscopy assessment of 3- and 6-week biofilm topography formed on human teeth (representative images) by *Enterococcus faecalis* (*E. f.*), *Candida albicans* (*C. a.*), and *Enterococcus faecalis/Candida albicans (E. f./C. a.*) with and without treatment with CSA-44. Preformed biofilm on human tooth surfaces after of grown was subjected to ceragenin CSA-44 treatment at a concentration of 20 µg/mL. CT−, teeth stored in a growth medium without inoculation; CT+, untreated biofilms (3 or 6 weeks) on teeth of the indicated microorganisms; 20 µg/mL, biofilms on teeth treated with CSA-44 (20 µg/mL) for 2 h. Images were collected using AFM working in Quantitative Imaging mode (QI by JPK) in wet conditions. The arrows indicate important details on the tooth surface as well as the morphology of healthy and damaged cells of the microorganisms tested. Orange arrows indicate bacterial cells, green arrows indicate fungal cells, the red arrows indicate cellular destruction under the action of CSA-44, and gray arrows highlight the tooth surface.

The morphology of the biofilm formed on teeth and the biofilm surface topography after treatment with CSA-44 were assessed using atomic force microscopy. The microscopic images provide additional information on the structure of the biofilms, demonstrate the ability of CSA-44 to inhibit biofilm growth, and show the nature of ceragenin action on the biofilms and on the morphology of individual bacterial and fungal cells. Figure 2 shows the morphology of 3- and 6-week biofilms of *E. faecalis*, *C. albicans,* and *E. faecalis*/*C. albicans* treated with ceragenin CSA-44 at a concentration 20 µg/mL on the surface of human teeth.

**Figure 3:**
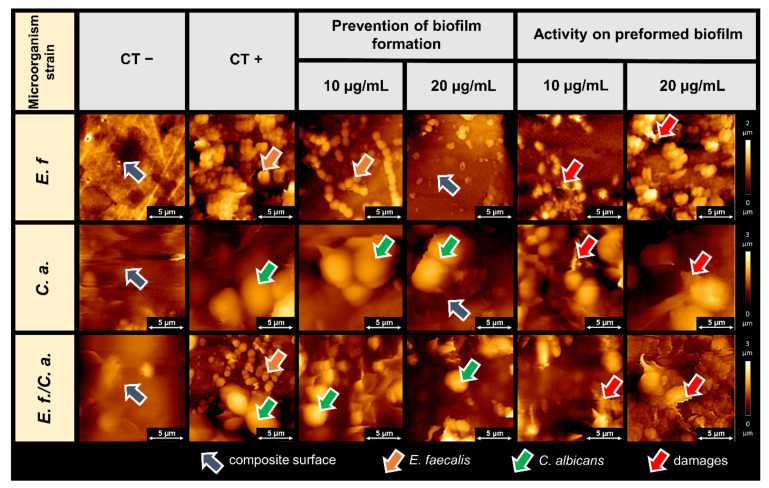
AFM topography of microbial biofilms on dental composites. CT−, composite material stored in a growth medium without inoculation; CT+, untreated biofilms (72 h) of the indicated organisms on the composite; Prevention of biofilm formation, images of composite surfaces after exposure to the indicated organisms in the presence of CSA-44 (10 or 20 µg/mL) for 72 h; Activity on preformed biofilm, images of established biofilms (72 h) of the indicated organism treated with CSA-44 (10 or 20 µg/mL) after 2 h of incubation. The arrows indicate important details within the images. Orange arrows indicate bacterial cells, green arrows indicate fungal cells, the red arrows indicate cellular destruction under the action of CSA-44, and gray arrows highlight the composite surface.

Figure 3 shows the topography of mature (72 h) biofilm formed on the surface of a dental composite and the preventive effects of CSA-44 on biofilm growth. Initially, we imaged the surface of control sample composites kept in growth medium (labeled as CT−) and dental composite samples with biofilm of *E. faecalis*, *C. albicans*, and *E. faecalis*/*C. albicans* (labeled as CT+). Gray arrows indicate the surface of the clean dental composite. The surface shows unevenness due to processing, typical of dental work during the cavity filling process. A developed biofilm was observed on the surface of the CT+ samples, which included *E. faecalis* cells (orange arrows) and *C. albicans* cells (green arrows). Images of the preformed biofilm after CSA-44 addition (final concentrations of 10 and 20 µg/mL) are presented. Representative pictures of preventative effects of CSA-44 show reduced numbers of bacterial and fungal cells. Areas of the exposed composite (with no biofilm growth) indicate the preventive effect of the applied ceragenin. Additionally, with higher concentrations of ceragenin, less biofilm adhered to the composite surface.

The effects of ceragenin CSA-44 on preformed biofilms on the composite surface were substantial. The topography images show destruction of the cells within the biofilm and changes in the organizational futures of the biofilm network. Cell destruction (e.g., outflows, cell perforation) is indicated by red arrows. AFM scan traces indicate decreased cell adhesion and changes in cell rheology.

**Figure 4:**
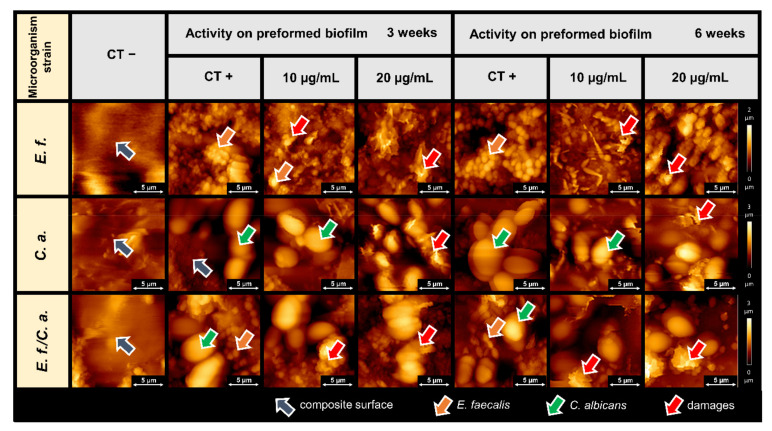
Morphology of the biofilm formed by *Enterococcus faecalis* (*E. f.*), *Candida albicans* (*C. a.*), and *Enterococcus faecalis/Candida albicans (E. f./C. a.*) at 3 and 6 weeks on a composite surface with and without CSA-44 addition. CT−, composite material stored in growth medium without inoculation; CT+, untreated biofilms (3 and 6 weeks) of the indicated organisms on the composite; Activity on preformed biofilm, images of established biofilms (3 or 6 weeks) of the indicated organism treated with CSA-44 (10 or 20 µg/mL) after 2 h of incubation.

Actions of CSA-44 on biofilms formed on the dental composite over long term (3 and 6 week) are shown in Figure 4. Topography assessment shows the nature of the surface of the non-inoculated composite (CT−), structures of established biofilms (CT+), and the destruction of biofilm-forming cells upon CSA-44 addition (10 and 20 µg/mL).

**Figure 5:**
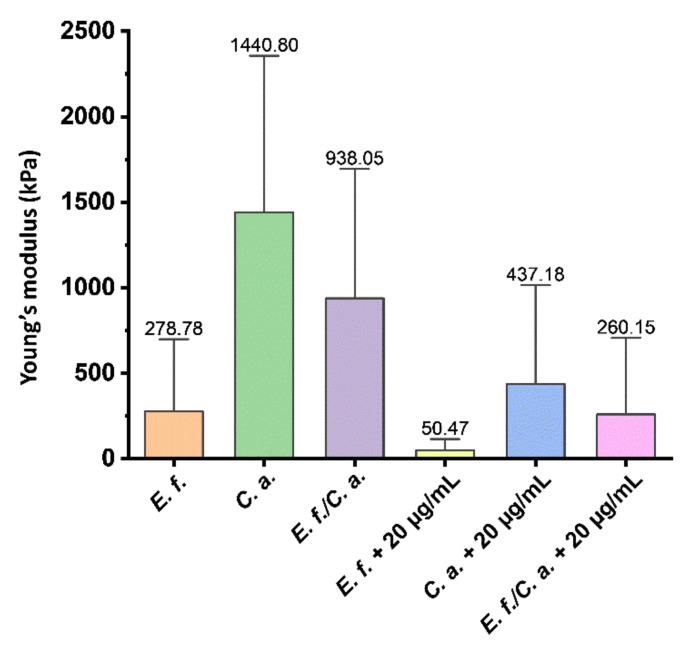
Stiffness of *Enterococcus faecalis* (*E. f.*), *Candida albicans* (*C. a.*), and *Enterococcus faecalis/Candida albicans (E. f./C. a.*) biofilm grown (6 weeks) on a dental composite before and after treatment with CSA-44 (20 µg/mL). First three bars indicating the Young’s modulus mean values of preformed biofilm composed of *E. faecalis*, *C. albicans*, and *E. faecalis/C. albicans* cells without ceragenin CSA-44 treatment. These biofilms were considered as control samples. The next bars show the changes in the stiffness of control preformed biofilms upon ceragenin CSA-44 addition at a concentration of 20 µg/mL. Young’s modulus values were measured using an AFM indentation technique. Force-indentation curves were collected using a silicon nitride cantilever with a polystyrene bead attached. The elastic modulus curves were fitted to the Hertz contact model.

The effect of ceragenin on the overall biofilm mass was characterized by studying the biofilm rheology with a dedicated AFM spherical tip. Figure 5 shows changes in biofilm rheological properties upon CSA-44 addition. A 6-week mature biofilm grown on the dental composite surface was evaluated. Modulus of elasticity values (Young’s modulus) of biofilm were obtained using an AFM indentation technique and calibrated on force-indentation curves. Among the control biofilms, the *C. albicans* biofilm had the highest modulus of elasticity 1.4 MPa, while the *E. faecalis* biofilm had the lowest modulus of elasticity of 0.3 MPa. In each case, after ceragenin addition, the elasticity of the formed biofilms decreased, which suggests an effect of the compound on the structures of the biofilm networks. After treatment, the stiffness of the *E. faecalis* biofilm decreased by 82%, that of the *C. albicans* biofilm decreased by 70%, and that of the mixed biofilm decreased by 72%.

**Figure 6:**
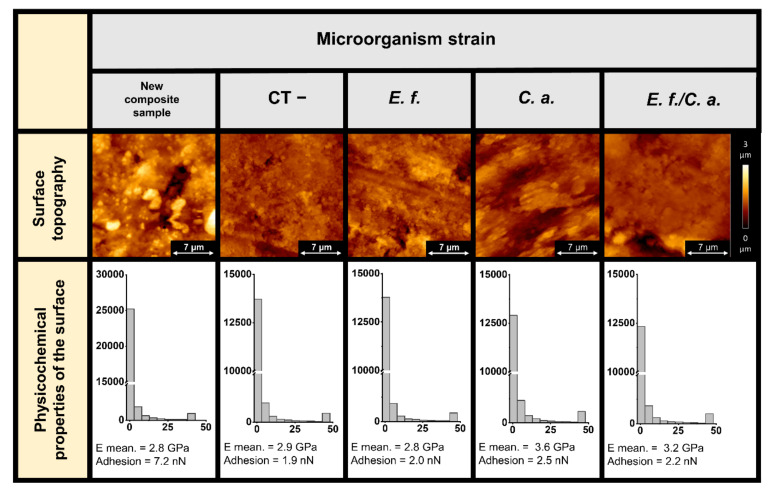
Effect of microorganisms on the surface characteristics of dental composites. Examination of AFM topography and visualization of stiffness (Young’s modulus, E) and adhesion of composite surfaces before and after biofilm removal. Measurements were carried out using preformed 6-week-old biofilm (*Enterococcus faecalis* (*E. f.*), *Candida albicans* (*C. a.*), and *Enterococcus faecalis/Candida albicans (E. f./C. a.*)). Images were collected using AFM working in Quantitative Imaging (QI) mode in wet conditions. Local mechanical properties (Young’s modulus, adhesion) of the new composite and composites after incubation and biofilm removal were calculated based on collected force-indentation curves (elasticity maps) taken from AFM microscope measurements. Young’s modulus of composites was derived from the Hertz–Sneddon model. In each column, the Young’s modulus values distributions and mean values of Young’s modulus and adhesion forces between AFM cantilever and composite surface are shown. Composite samples stored in growth medium without microorganisms are labeled as CT−. All changes in physical properties were compared to a new sample (new composite sample).

Additionally, the effect of microorganisms on the surface futures of dental composites was investigated. AFM topography and the values of Young’s modulus and adhesion of composite surfaces before and after biofilm removal are presented in Figure 6. Surface alterations that might develop on dental composite upon microorganism growth were investigated as well. Figure panels of the surface topography of the composites after biofilm removal using sonication do not indicate a definite influence of biofilm on the polymer structure. No obvious damage in the form of micro-cracks or other mechanical destruction was observed, while studies of the physicochemical properties of the surfaces indicated changes in properties after biofilm formation and removal. The Young’s modulus of the surface on which the *C. albicans* biofilm grew increased from 2.8 to 3.6 GPa. Furthermore, the mixed biofilm, in which the *C. albicans* was also present, caused an increase of stiffness of the dental composite surface. Growth of the bacterial biofilm did not increase the stiffness. Surface adhesion decreased in each case. The Young’s modulus distributions indicate two mechanical phases in the composite structure. In the area of the tested material, there are zones of increased stiffness, probably related to the presence of the biofilm matrix.

## 3. Discussion

Since the formation of biofilm on teeth and dental fillings, typically made of composite materials, is a serious threat that can result in the development of caries and various periodontal diseases, we examined the potential of ceragenin CSA-44 to target mature biofilms and its ability to prevent biofilm formation in vitro on the surfaces of extracted teeth and composite discs. We optimized our experimental conditions to closely mimic the natural environment in which oral biofilm develop by integrating human saliva (50%) into the medium supporting biofilm culture and subjecting the composite material to the mechanical stresses that are usually induced during chewing before culturing of the preformed biofilm was begun. CSA-44 activity was tested against *E. faecalis* because it has been previously described as a bacterium difficult to eliminate, especially during root canal therapy, and *C. albicans* because it is an opportunistic fungus that very often becomes pathogenic when the oral environment is disrupted as a result of antibiotic therapy [21,22]. It is worth noting that *C. albicans* has virulence factors that enable it to adhere to and colonize mucous membranes as well as implants and dentures, tooth fillings, root canals, or dental cements. It also has the ability to change its morphological form from yeast to hyphae. Different steps account for *Candida* biofilm development: adhesion, initiation (cells take a filamentous form), and then mature biofilm. The last phase is dispersion: the release of more virulent *Candida* cells from the biofilm into the surrounding environment. It has been noted that for patients who use removable prosthetic restorations, it is necessary to repair or replace a restoration made of acrylic after *C. albicans* infection [23,24,25,26]. The question is still unclear whether similar procedures should be performed for composite fillings and filled root canals. The biofilm present in the human oral cavity in vivo is a mixture of different microorganisms, and the elimination of biofilm in certain clinical conditions that affect an oral cavity may be the only way to avoid pathological consequences of biofilm development.

The preventive effect of ceragenin CSA-44 on biofilm adhesion to teeth and composite surfaces was evaluated using AFM. AFM topography provides valuable information on the changes occurring in the structure of biofilm-forming cells under the influence of bactericidal compounds. These changes were previously observed for human antibacterial peptides, their synthetic analogues, and some nanosystems [20,27,28,29,30,31]. Studying the biofilm surface has been previously explored to provide valuable information regarding biofilm mass and the antimicrobial activity of tested compounds [29].

In the present study, the antibiofilm activities of CSA-44 were assessed in a similar manner. We observed that treated biofilm samples had fewer visible clusters of biofilm structures. The effect of the ceragenin on biofilm formation was also evident based on the observed destruction of bacterial and fungal cells. Cell lysis is manifested by damage to the cell membranes and outflow of the cell interior. A change in the cell shapes were visible as well as the increase in cell adhesion parameters. These are typical ceragenin effects on the microbial cell membranes, resulting in membrane perturbation, as was previously reported [32,33,34,35]. It is worth underlining that the here-observed ability of CSA-44 to decrease adhesion of developed biofilm indicates the ability of this agent to maintain its activity within the network of negatively charged biopolymers that compose the extracellular matrix of biofilm. However, it is unclear whether the molecular action of CSA-44 requires direct, charge-dependent interaction with these biopolymers and whether it is regulated by charge distribution in CSA-44 molecules.

Interestingly, the stiffnesses of biofilms formed by *E. faecalis*, *C. albicans,* and *E. faecalis/C. albicans* differ from each other, suggesting that stiffness may be a species-specific feature. The biofilm formed by *C. albicans* cells was stiffer than that from *E. faecalis.* A large part of the biofilm structure stiffness originates from the stiffness of the cells themselves, which might be considered as elastic, deformable spheres [36,37]. In a previous report [35], the Young’s modulus of *Bacillus subtillis* cells surface was determined to be about 800 kPa. Others [38] determined the Young’s modulus of fungal cells to be up to 760 kPa, but a different report [36] estimated the Young’s modulus of *C. albicans* cells as only 360 kPa. Based on these conflicting reports [39], it is also not possible to state unequivocally which cells are stiffer when measured using AFM microscopy. The stiffness appears to depend on the type of cell, the type of substrate on which the biofilm is formed, and the external conditions. However, we can conclude that in each case, extracellular polysaccharide (EPS) affects the stiffness of the biofilm in only a small way.

In the case of our measurements, the shape of the AFM tip was of great importance. Given the diameter of the sphere at the tip and the size of the bacterial cells, the cells may have been more susceptible to deformation and displacement together with the EPS. Taking into account the geometry of the AFM tip, a biofilm that contained larger fungal cells may have had higher stiffness.

The purpose of this aspect of the study was to measure the change in stiffness of the formed biofilm under the influence of CSA-44. The decreases in Young’s modulus that we recorded indicate that the biofilm was loosened upon CSA-44 treatment. An additional part of the study was to determine the effect of the formed biofilm on the physical properties of the dental composite surfaces. Our results indicate that the biofilm composed of *C. albicans* cells caused a local increase in the stiffness of the dental composite material. Similar changes in mechanical properties due to biofilm influence have been reported for polymeric vocal prostheses [31]. Additionally, microbial cells were found to grow inside the polymers and caused cracking. Another study proved that biofilm can damage even hard metallic biomaterials [40]. Considering the influence of all biofilm components, enzymatic degradation was identified as one of the pathways toward biomaterials degradation in the oral cavity [40,41,42,43,44]. When biofilm is present, changes in material mechanics caused by the presence of microorganisms can also proceed locally, causing heterogeneities in material structure and mechanics [31,45]. Heterogeneities in the physical properties of the material (as indicated by recorded changes in Young’s modulus and surface adhesion), which are not visible in AFM topography images, can cause unexpected accumulations of adverse stresses under cyclic loading and differences in thermal conductivity causing thermal stress. This condition can cause micro-cracking of the material [45,46], which in the long run, can cause macroscopic cracking and material failure [45,46,47,48].

## 4. Materials and Methods

### 4.1. Collection of Human Tooth and Fabrication of Composite Disc

A collection of 60 natural, single-root teeth from healthy patients removed for orthodontic and occlusive indications were used. Extractions were performed under local anesthesia, according to the atraumatic removal procedure (protocol approved by the Ethics Committee in Research of Medical University of Bialystok, IRB approval: R-I-002/178/2019). After extraction, teeth were cleaned with 30% hydrogen peroxide and wiped with a sodium hypochlorite solution (6 % NaOCl, CanalPro COLTENE). Before tests, teeth were stored in PBS medium. In addition to human teeth, dental composites were been prepared. Dental composites are the most popular material in modern dentistry, consisting of a polymerizable resin matrix, reinforced glass particle fillers, and silane coupling agents [46]. A set of 25 round-shaped discs (10 mm diameter, 6 mm thickness) were fabricated using conventional light-cured composites according to manufacturers’ instructions [49]. Dental composite discs were prepared using a polymethyl methacrylate mold in the form of two-piece plates with holes of appropriate dimensions, screwed together during polymerization. After polymerization, the surfaces of the produced dental composite discs were polished with a finishing bur to achieve a surface roughness compatible with standard dental procedures. Next, the dental composite samples were aged for 3 months [46] and daily brushed with a standard toothbrush with a popular commercial toothpaste to imitate conditions in the human oral cavity. In an oral environment, dental composites may undergo material property changes due to mechanical factors, such as toothbrushing, wear by chewing, and abrasion. In order to mimic the mechanical stresses present in the oral cavity, which can cause micro-cracking and wear of dental materials in normal use, composite samples at the end of the aging time were subjected to fatigue wear using a friction machine for mimicking chewing forces. Courtesy of the Faculty of Mechanical Engineering of the Białystok University of Technology, a pin-on-disc friction tester was used. The counter sample in pin-on-disc tester was an Al_2_O_3_ ceramic disc. The ceramic disc was rotated at a constant speed of 50 rpm, while the front surface of the composite was pressed against the disc with a constant force of 100 N. The treatment was carried out in a saline solution of 0.9% NaCl for 1 h for each sample, which is a friction distance of about 1.88 × 105 mm, a condition that corresponds to the pressure in the mouth during chewing. After fatigue cycling and before biological testing and AFM research, each disc or tooth was placed in a 6- or 12-well culture plate under sterile conditions. Tested dental composite discs were attached (worn surface up) inside the wells using bonding resin, whereas teeth were attached using hard wax. UVC lamp was used for sterilization. A portion of filtered saliva prior to biofilm development was added to each well as required. Saliva was collected from a healthy volunteer [49] who afforded written formal acceptance according to a protocol approved by the Ethics Committee in Research of Medical University of Bialystok (IRB approval: R-I-002/178/2019).

### 4.2. Strains of Microorganism

*E. faecalis* ATCC 29212 and *C. albicans* ATCC 26790, obtained from ATCC-American Type Culture Collection, were used in this study. The tested strains were cultured and maintained on the recommended media, i.e., blood agar with the addition of 5% defibrinated sheep blood (BioMaxima, Poland) for *E. faecalis* ATCC 29212 and Sabouraud Dextrose agar with the chloramphenicol (BioMaxima, Poland) for *C. albicans* ATCC 26790.

### 4.3. Ceragenin

CSA-44 was synthesized as described previously [50] and afterwards dissolved in phosphate-buffered saline (PBS, Thermo Fisher Scientific, Waltham, MA, USA). Prepared solutions of CSA-44 were stored in 4 °C.

### 4.4. Prevention of Biofilm Formation

The strains of *E. faecalis* and *C. albicans* in logarithmic growth phase cultured on appropriate media (blood agar with the addition of 5% defibrinated sheep blood or Sabouraud Dextrose agar with the chloramphenicol addition) were harvested and suspended at a population of 10^8^ CFU/mL in the mixture of Luria–Bertani broth (Biomaxima, Poland) and filtered patient’s saliva in a 1:1 ratio. Prepared suspensions of microorganisms (*E. faecalis*, *C. albicans,* and *E. faecalis/C. albicans* in a ratio 1:1) were added to 6-well, white-bottom plates with CSA-44 at concentrations of 10 and 20 µg/mL. Dental composites, prepared as described above, were added to the wells. The culture was maintained at 37 °C in aerobic conditions for 72 h. A medium (the mixture of Luria–Bertani broth and filtered patient’s saliva in a 1:1 ratio) was changed every single day before adding the next dose of CSA-44. To determine the antibiofilm properties of CSA-44, crystal violet (CV) staining (0.1%) was performed. After incubation, the plates with the dental composites were washed with PBS to remove the unadhered microorganisms. Then, plates were stained using the 0.1% crystal violet (Sigma-Aldrich, Saint Louis, MO, USA) for 15 min at room temperature. Excess stain was removed, and dental composites were carefully rinsed using deionized water and left to dry. To solubilize the crystal violet, 98% ethanol was added for 15 min at room temperature. Finally, the optical density of the dye attached to stained biofilms was measured at a wavelength of 580 nm using a Labsystem Varioscan Lux (Thermo Fisher Scientific, Waltham, MA, USA).

### 4.5. Disruption of Established Biofilms

Single colonies of *E. faecalis* and *C. albicans,* in mid-log phase growth from an overnight culture in an appropriate medium at 37 °C in aerobic conditions, were suspended in sterile Luria–Bertani broth mixed with filtered human saliva (1:1). Cell densities of microorganisms were adjusted to 10^8^ cells/mL. Biofilms of *E. faecalis*, *C. albicans,* and *E. faecalis/C. albicans,* in a proportion 1:1, were grown in 6-well white-bottom plates with (i) teeth for 3 or 6 weeks or (ii) dental composites for 72 h or 3 or 6 weeks. The culture was maintained at 37 °C in aerobic conditions through the end of required incubation time, and then, CSA-44 was added in concentrations of 10 and 20 µg/mL. After exposure for 2 h, plates were washed 3× with PBS to remove planktonic cells. The mass of biofilm was investigated using crystal violet staining (0.1%) following a previously mentioned protocol.

### 4.6. Evaluation of Antibiofilm Activity-AFM Measurements

Topography and rheological properties of dental composites, teeth, and biofilms on surfaces were measured using an atomic force microscope (AFM) NanoWizard 4 BioScience AFM (JPK Instruments, Bruker USA) equipped with a liquid setup. Due to the lateral forces that occurred during topography measurements between AFM probe and biofilm, force curves-based imaging mode JPK QI™ (Quantitative Imaging mode) was used. Topography maps size of 10 μm × 10 μm and 5 μm × 5 μm were carried out with resolution of 128 pixels per line to show characteristic of the dental composite material surfaces and biofilm morphology. *E. faecalis*, *C. albicans,* and *E. faecalis/C. albicans* biofilms formed on dental composite were measured after 72 h and 3 and 6 weeks of growth. Biofilms formed on teeth were measured after 3 and 6 week of growth. Surfaces of composite materials after 6-week biofilm removal were also evaluated. Biofilm topography maps were collected using a silicon nitride cantilever Bruker MSCT (Bruker, Billerica, MA, USA), with a nominal spring constant of 0.07 N/m and measured spring constant in the range of 0.06–0.09 N/m. For cantilever calibration, the thermal tune method was used. Topography maps of dental composite material were collected using a silicon nitride cantilever HQ:NSC36/NoAl MikroMasch (NanoAndMore GMBH) with a nominal spring constant of 0.6 N/m and spring constant in the range of 0.5–0.7 N/m measured using the thermal tune method. In this case, QI maps were also used to determine composite surface elastic modulus (i.e., the Young’s modulus) and adhesion forces between composite surfaces and the AFM probe.

To determine the Young’s modulus, measured force-indentation curves were fitted to the Hertz–Sneddon contact model. Histograms of the Young’s modulus value distributions for each composite materials were prepared. The mean values of Young’s modulus as well as adhesion forces between AFM probe and composite material surfaces were also calculated. For biofilm (formed on composite materials) stiffness measurements, force-indentation curves were collected on a stiff substrate and on the elastic biofilm. Due to the overall biofilm mass high deformability, force-indentation curves were collected using a silicon nitride cantilever with a nominal spring constant of 0.2 N/m and spring constant in the range of 0.15–0.3 N/m measured using the thermal tune method, with a 4.5 μm diameter polystyrene bead attached. The cantilevers were manufactured by Novascan Technologies USA. Up to 5 force-indentation maps consisting of 16 × 16 points corresponding to a scan area of 50 × 50 μm were made for each sample. Maps were taken from multiple places on composites. After separation of the force-indentation curves recorded on the clean composite material surface and on the biofilm structures, we obtained properties of biofilms. Mean values ± standard deviation for biofilms treated using CSA-44 and untreated were calculated. All AFM experiments were made 1 h after sample preparation in wet conditions at room temperature. During the examination, teeth were glued into Petri dishes upright. Tooth biofilm topographies were measured from the tooth crown. The composites were also placed in the petri dishes and tested in wet conditions at room temperature.

### 4.7. Statistical Analysis

The significance of differences was determined using the two-tailed Student’s *t*-test. Statistical analyses were performed using Graph Pad Prism, version 8 (San Diego, CA, USA). * indicates statistical significance at ≤ 0.05, ** ≤ 0.01 and *** ≤ 0.001.

## 5. Conclusions

The ability of ceragenin CSA-44 to prevent the formation and adhesion of biofilm to surfaces of tooth and composite filling surfaces indicates that CSA-44 may be a molecule of use for combating oral pathogens that cause negative effects, including oral cavity infections. Ceragenins may serve as new ingredients in oral care products.

## Data Availability

The data that support the findings of this study are available from the corresponding author upon reasonable request.
